# Recent Progress in Nonconventional Luminescent Macromolecules and their Applications

**DOI:** 10.1021/acs.macromol.4c00186

**Published:** 2024-06-11

**Authors:** Nan Jiang, Chang-Yi Zhu, Ke-Xin Li, Yan-Hong Xu, Martin R. Bryce

**Affiliations:** † Key Laboratory of Preparation and Applications of Environmental Friendly Materials, Key Laboratory of Functional Materials Physics and Chemistry of the Ministry of Education, Jilin Normal University, Changchun, 130103, China; § Department of Chemistry, Durham University, Durham DH1 3LE, U.K.

## Abstract

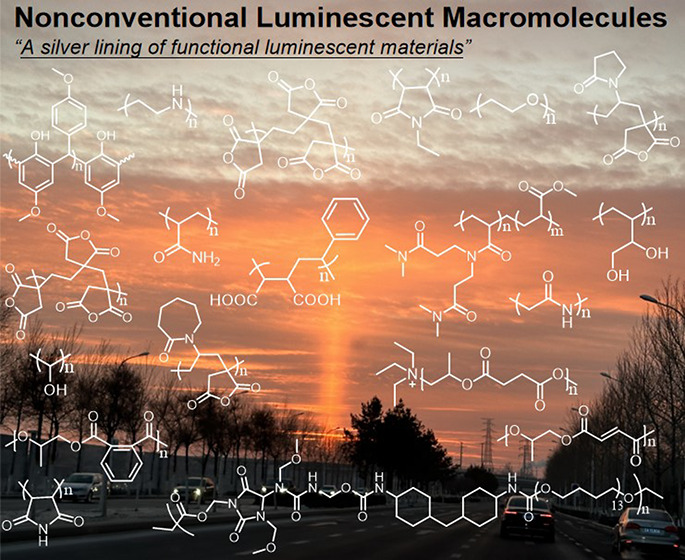

Traditional π-conjugated luminescent macromolecules typically suffer from aggregation-caused quenching (ACQ) and high cytotoxicity, and they require complex synthetic processes. In contrast, nonconventional luminescent macromolecules (NCLMs) with nonconjugated structures possess excellent biocompatibility, ease of preparation, unique luminescence behavior, and emerging applications in optoelectronics, biology, and medicine. NCLMs are currently believed to produce inherent luminescence due to through-space conjugation of overlapping electron orbitals in solid/aggregate states. However, as experimental facts continue to exceed expectations or even overturn some previous assumptions, there is still controversy about the detailed luminous mechanism of NCLMs, and extensive studies are needed to further explore the mechanism. This Perspective highlights recent progress in NCLMs and classifies and summarizes these advances from the viewpoint of molecular design, mechanism exploration, applications, and challenges and prospects. The aim is to provide guidance and inspiration for the huge fundamental and practical potential of NCLMs.

## 1 Introduction

Organic luminescent macromolecules (OLMs) have the advantages of good structure–property adjustability, synthetic flexibility and biocompatibility. Their wide scope of applications in advanced fields such as sensors, medicine, catalysis and optoelectronic devices has been an important and persistent topic.^1−5^ However, the current molecular design principles of traditional OLMs have relied upon the strong donor and acceptor functional groups with large π-coupled substructures [such as double bond, triple bond or (hetero)aromatic ring].^6−9^ Such macromolecules tend to have poor water solubility, are nonbiodegradable and can be biotoxic; in addition, they often require expensive raw materials and complex, time-consuming, synthetic protocols involving potentially toxic heavy-metal catalysts and extensive purification to remove these catalyst residues. Moreover, these conventional materials are often severely affected by aggregation-caused quenching (ACQ) due to the inevitable π–π packing interactions. The emission efficiency in the aggregated/solid state is therefore usually very low, which greatly hinders the development of OLMs in practical applications, unless they are dispersed in specific host materials.^10−12^ In 2001, Tang et al. promoted the concept of Aggregation-Induced Emission (AIE) in the aggregated state which is the most common form of luminescent materials in practical applications.^13^ OLMs with AIE characteristics (AIE-OLMs) show great potential in optoelectronic devices, chemical sensing, biological detection and imaging diagnosis and treatment.^14,15^ However, the emission from traditional AIE-OLMs is based on a mechanism of restricted molecular motion, which limits the structural diversity of AIE molecules to a certain extent. Therefore, breaking out of the inherent framework of existing AIE molecular design and developing new AIE-OLM systems will undoubtedly expand the diversity of AIE molecules, uncover new luminescence mechanisms and expand the horizon for new applications.^16,17^ The involvement of electron-rich, heteroatomic subluminophore (HASL) moieties has been illustrated and described in at least four specific types of molecular level (spacial/steric) confinement events: namely (i) architecture (i.e., dendritic); (ii) chemical cross-linking (i.e., rigidity); (iii) supramolecular (i.e., clustering); and (iv) physical (i.e., pressure) that are associated with essentially all known examples of nonconventional intrinsic luminescence in dendrimers, macromolecules and small molecular structures that lack conventional luminophores.^16^ This extensive review by Tomalia and co-workers is widely recognized as an important rationalization that unifies nonconventional luminescence starting from the first observations in the 1970s.

The present perspective article will focus on nonconventional luminescent macromolecules (NCLMs) that lack well-defined conventional chromophores or π–conjugated structures, and they also exhibit enhanced emission due to limited intramolecular migration, which is similar to AIE behavior.^18−20^ Examples include polyethers, polyesters, proteins, cellulose and its derivatives, polyurethanes, oligo(maleic anhydride)s, polyisobutene derivatives, polysiloxanes and so on.^21−28^ It is generally agreed that despite the absence of large aromatic conjugated structures, the sharing and overlap of electron clouds of functional groups such as hydroxyl (−OH), ester (−COOR), carboxyl (−COOH), carbonyl (C=O), alkene (C=C), sulfoxide (S=O), ether (−O−), amide (−NHCO−), amine (−NH_2_), cyanide (C≡N), thioether (−S−), or subgroups containing halogens (Cl, Br, and I) can provide through-space conjugation (TSC) by electron overlap from nonbonded atoms which extends electron delocalization and achieves a rigid molecular conformation. Such multiple intramolecular and intermolecular interactions can effectively inhibit quenching effects and nonradiative relaxation, which will contribute to the inherent singlet and triplet luminescence (i.e., fluorescence and phosphorescence) of NCLMs.^29−31^ Benefiting from these simple chemical structures, compared with traditional AIE-OLMs, NCLMs have low raw material cost, simple synthesis, easy large-scale production, good biocompatibility, and a wide range of proven applications, notably in sensing, optoelectronic displays, anticounterfeiting and biomedical fields.^32,33^

However, the detailed mechanism behind the emission of NCLMs remains a puzzle that has hindered their development compared to the traditional AIE-OLMs based on through-bond conjugation (TBC).^34−36^ Although NCLMs have been widely reported over the past 20 years, their structure–property relationships and their anomalous emission mechanism have remained controversial and are constantly being revised.^37,38^ For example, it was generally understood that NCLMs are usually accompanied by concentration-dependent emission and excitation-dependent luminescence. However, many nontraditional luminophores have been found that do not have these characteristics.^23,39^ Many fundamental questions are being asked. For examples: (i) How can the real launch center of the emission be determined? (ii) When individual molecules come together how do their interactions affect the NCLMs’ macroscopic properties? (iii) How can NCLMs’ photophysical properties be regulated at the molecular level? These problems are still the key and difficult issues to be explored in this field.

In addition, due to the limited understanding of the detailed mechanism of NCLM emission the rational modulation of luminescence over the entire visible range remains difficult.^40,41^ The initial challenges were limited to blue and green emission, and to overcome low photoluminescence quantum yield (PLQY).^42,43^ To date, achieving both long-wavelength emission (yellow, red, and even near-infrared (NIR)) and high PLQY from such systems remains challenging.^44−46^ For organic fluorescent materials with conventional TBC the emission wavelength and efficiency can be modulated by increasing the π-conjugation and by introducing π– donor and π– acceptor (D-A) units.^47−49^ But how can the luminescence of NCLMs be regulated? Current strategies focus on the following aspects. At the molecular level: (i) Introducing different heteroatoms and electron acceptor/donor functional groups to change the electronic structure of subunits, thus promoting charge transfer (CT) and electron delocalization;^43^ (ii) Changing the chromophores’ steric confinement to make the polymer more rigid which limits the movement of the polymer chains;^50,51^ (iii) Increasing the number of electron-rich units, thereby enhancing electronic communication within and between molecules, thus affecting the wavelength and efficiency;^52^ (iv) Introducing stronger molecular interactions, such as coordination bonds, hydrogen and ionic bonds, which also induce the aggregation and luminescence behavior;^53,54^ (v) Adjusting the external environment, (e.g., temperature, pressure, pH, the addition of salts and urea in aqueous solutions) to affect the conformational rigidity and the electronic communication.^55,56^ At the macromolecular level recent studies have shown that chain structures that are too flexible or too rigid can destabilize the clusters due to variable segmental mobility, making it difficult to control the polymeric structure and to form stable intra/intermolecular interactions, so the luminescence will be weakened. However, a moderately rigid chain structure can optimize the segmental motion, which is conducive to the formation of more uniform and stable clusters, thereby enhancing the luminescence of the polymer chain. Therefore, balancing the structural flexibility and rigidity of NCLMs is also a feasible strategy to improve the photophysical properties of NCLMs, such as emission wavelength and efficiency.^57,58^

The above strategies have been partly successful in the manipulation of the luminescence properties of NCLMs. However, precisely regulating the photophysical properties of these flexible NCLMs at the molecular level remains a great challenge. To construct a more comprehensive map of the mechanism of nontraditional luminescence, extensive experimental and theoretical work is still needed to deeply explore the structure–property relationships of NCLMs. This is a prerequisite for developing their advanced applications. Based on the above background, this Perspective highlights examples of the molecular and macromolecular design principles, the developments of the aggregate luminescence mechanism, and the applications of NCLMs since 2020. The future research focus and prospects for NCLMs are also discussed. Our aim is to inspire the construction and functional development of organic aggregates with enhanced luminous performance and to bring more NCLMs from the laboratory to the world of commercial applications.

## 2 Macromolecules with Nonconventional Luminescence

### 2.1 In-Depth Exploration of Blue Light Emission

In the early 2000s, most of the known NCLMs were blue emitters.^16^ Indeed, the continued development of blue NCLMs remains of great significance in finding simple and efficient synthetic methods, establishing the structure of the true luminescence center, understanding the luminescence mechanism, and predicting and regulating their photophysical properties.

**Figure 1:**
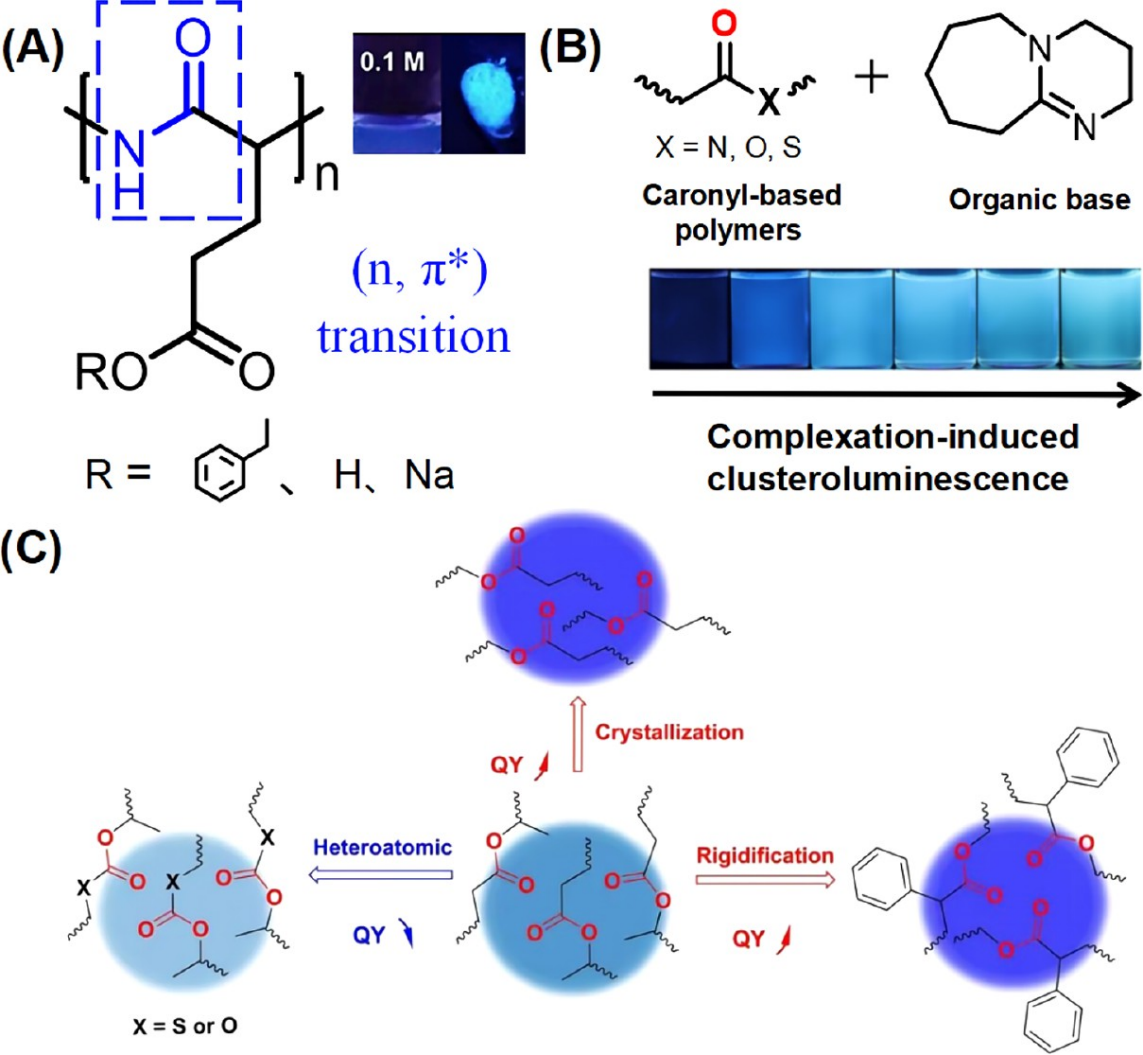
(A) Chemical structures of three polypeptides. Inset: Photos of them in dilute solution and solid state. Reprinted with permission from ref (22). Copyright 2022, Elsevier.^22^ (B) Top: chemical structures of carbonyl-based polymers and DBU; Bottom: photos of the complexes between carbonyl-based polymers and DBU taken under 365 nm UV lamp as mixing time goes on.^60^ Reprinted with permission from ref (60). Copyright 2023, Wiley-Blackwell. (C) Schematic diagram of the effect of crystallization, addition of heteroatoms and increase of rigidity on the luminescence properties of NCLMs.^50^ Reprinted with permission from ref (50). Copyright 2022, Wiley.

In 2022, Tang et al. synthesized three nonconjugated polypeptides (Figure 1A), which do not have excitation-dependent luminescence properties.^22^ It was shown that fluorescence comes from the “chromophore” with a definite structure. Because the side chain substituents of the three polypeptides are different, it was speculated that the fluorescence may come from the main chain. Photophysical data and theoretical calculations demonstrated that the emission of these polypeptides and their template small molecules all derive from the (n-π*) transition of a single amide unit, rather than amide clusters. In addition, the authors summarized different classes of NCLMs with C=O groups, such as polyketones, polyesters, and polyamides, and found that they all emit at around 440 nm.^59^ Analysis of different types of nonconventional luminescent materials, including polymers and small molecules, concluded that this emission comes from carbon-based (n-π*) transitions. In addition, although fluorescence is derived from a single amide, intermolecular interactions such as hydrogen bonds and electrostatic interactions, as well as intramolecular hydrogen bonds, affect the efficiency of fluorescence through resulting conformational transformations.

A report in 2023 of the complexation-induced cluster luminescence of carbonyl polymers with nitrogen-rich organic bases (Figure 1B) revealed that the complexation not only enhanced the intrinsic 440 nm emission of the carbon groups due to the (n-π*) transition, but also induced a new long-wavelength fluorescence corresponding to the complex.^60^ This work proved that complexation has potential as a new strategy to regulate the luminescence of NCLMs. Studies on six nonconjugated carbonyl polymers with different heteroatoms and steric confinement also showed that the 440 nm emission is produced by the (n-π*) transition of the carbonyl group, and the emission strength is influenced by the electronic structure and conformational flexibility of the subunits. In general, high flexibility of the polymer chain weakens luminescence, and a relatively rigid polymer chain favors strong luminescence (Figure 1C).^50^

In 2022, Zhao et al. copolymerized the strong hydrogen bond donor acrylic acid with the strong hydrogen bond acceptor vinylcaprolactam to prepare a stimuli-responsive thermosensitive polymer with deep-blue cluster luminescence and upper critical solution temperature in aqueous solution.^3^ Wan et al. prepared a series of nonconjugated poly(1,3-dicarbonyl)s by nucleophilic substitution polycondensation with high yields (up to >99%) under mild conditions. Deep-blue emission was observed in the solid state. Compared to traditional polycondensation protocols this nucleophilic polycondensation method has the advantages of a self-accelerating effect and flexible stoichiometry of the monomer units, thereby expanding the library of monomers, methods, chemical structures, and luminescent properties.^61^ Mori et al. used reversible addition–fragmentation chain transfer polymerization to synthesize block copolymers and random copolymers based on nonconjugated vinyl amine and *N*-acryloyl-l-threonine.^56^ The research found that the emission intensity of a block copolymer is influenced by the pH of the aqueous solution, which leads to different clustering of the poly(vinyl amine) (PVAm) segments. In contrast, no significant pH-dependence in emission intensity was detected in random copolymers. Drop-cast films and powder samples of the block and random copolymers showed blue emission, compared to greenish-blue emission of the PVAm homopolymer.

Cloutet et al. investigated the polychromatic photoluminescence of poly(dihydropyran).^62^ It was again shown that oxygen aggregation caused by the restricted polymer conformation contributes to the luminescence. Han et al. found that the emission wavelength of hyperbranched polysiloxane in aqueous solution was closely related to the length of the alkane chain from 2 to 6 CH_2_ units: shorter alkane chains produced relatively longer wavelength emissions and high quantum yields (16–18%). Aggregation was ascribed to hydrogen bonding and amphiphilicity.^63^ The further exploration of blue NCLMs will help to reveal the luminous source of NCLMs more comprehensively and will also guide the design of NCLMs with excellent photophysical properties, broadening the horizon for new materials and applications.

### 2.2 Modulation of Long Wavelength Emission

Due to the lack of large π-conjugated units only a few NCLMs have green, yellow or red emissions. Many attempts have been made in the past few years to achieve longer emission wavelengths. Foremost examples will now be considered.

#### 2.2.1 Through-Space Charge Transfer (TSCT)

TSCT in molecules where emission comes from charge transfer by a through-space pathway between donor and acceptor units that are physically separated by a nonconjugated backbone has been used in the development of novel NCLMs.^64,65^ Regulating the spatial interaction between donor and acceptor units by varying their strength and planarity, their interaction distance and relative orientation, and using multiple donor/acceptor structures, has meant that the luminous color and efficiency can be adjusted. For example, Zhang et al. found that the linkage pattern in poly(maleimide) chains has a strong influence on their properties. Emission colors were tuned across the visible region by changing the polymerization conditions (free radical or anionic). Charge transfer is not favored along an electron-deficient C–C backbone, which is not conducive to the close packing of molecular chains. In contrast, for chains with repeating −C-N units, the electron clouds can be alternately distributed to form a continuous A-D-A-D sequence, which is conducive to tight packing and aggregation between molecular chains, thus bringing about red-shifted emission.^39^ Zhang et al. synthesized amine-capped polyesters with NIR luminescence through copolymerization induced by organic amines. The highly efficient and controllable blue-to-NIR NCLMs were realized through the structural effects of polymer chains relative to model small molecule mixtures and the TSCT between esters and amines.^36^

#### 2.2.2 Through-Space Interactions (TSI)

Enhancing strong and stable interchain and/or intrachain TSI (which may involve TSCT, but also includes hydrogen bonds and coordination bonds) is an effective way to prepare NCLMs with strong, red-shifted emission. TSI can enhance the intramolecular and intermolecular electronic communication and extend electron delocalization, thus affecting the wavelength and efficiency of NCLMs; it may also reduce Δ*E*_ST_ thus promoting reverse ISC (RISC), bringing long lifetime luminescence, which benefits the development of NCLMs. Common strategies for enhancing TSI are the optimization of a hyperbranched structure, the introduction of multiple unconventional chromophores, introduction of electron donor/acceptor groups, and the introduction of rigid chromophores (e.g., benzene ring/double bond) to balance the rigidity and flexibility of the chain.^66^ Based on the above strategies, initial achievements have been reported in the past few years.

**Figure 2:**
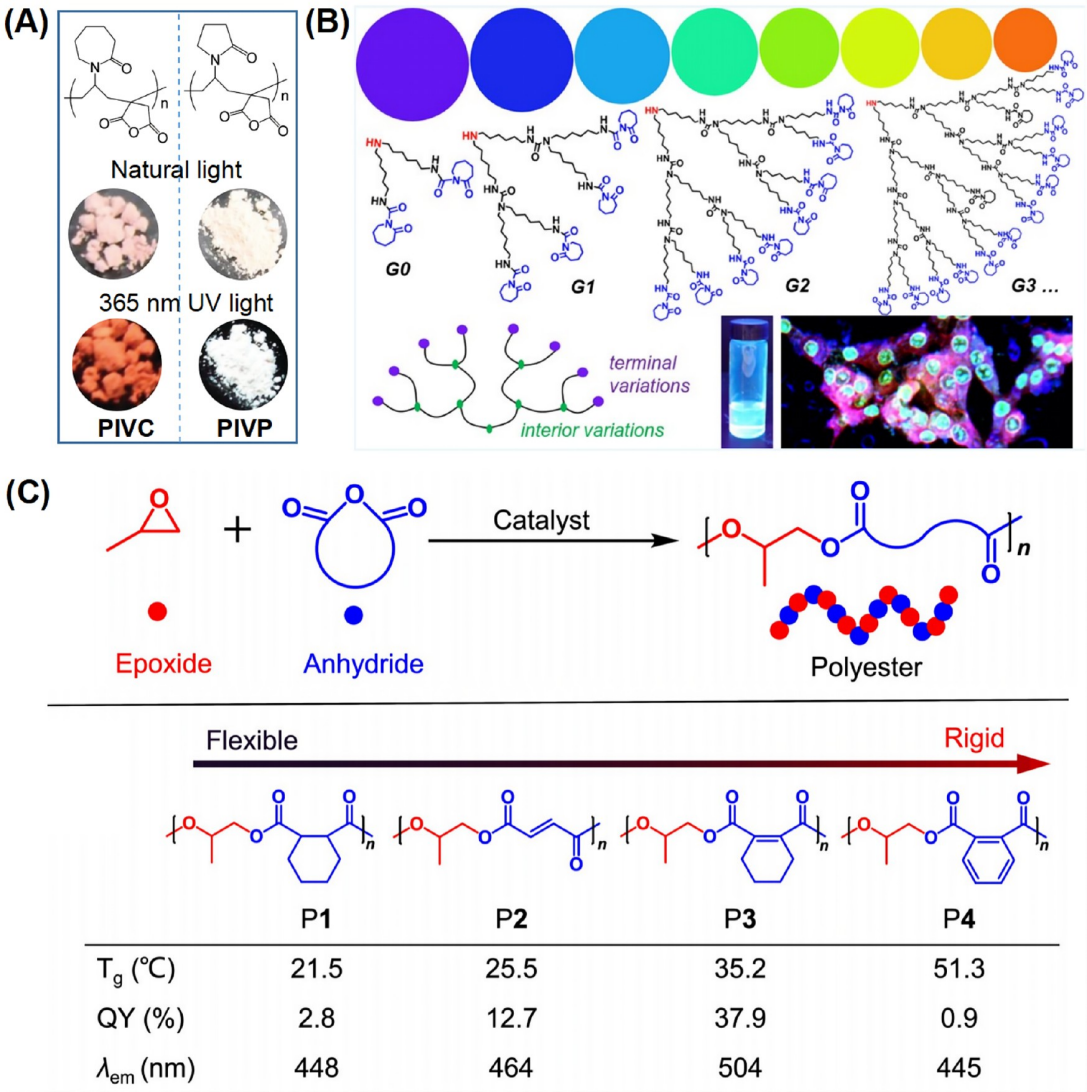
(A) Chemical structures of PIVC and PIVP; Photographs of PIVC and PIVP solid powders under natural light and 365 nm UV light.^57^ Reprinted with permission from ref (57). Copyright 2020, Royal Society of Chemistry. (B) Chemical structures, photographs, and *in vitro* imaging of aBPUs.^67^ Reprinted with permission from ref (67). Copyright 2022, American Chemical Society. (C) Chemical structures of P1, P2, P3, P4, listed in the increasing order of *T*_g_, indicative of the segmental mobility from flexibility to rigidity, summarized thermodynamic data for four polyesters. *T*_g_: glass transition temperature; λ_em_: PL maximum.^58^ Reprinted with permission from ref (58). Copyright 2022, John Wiley and Sons.

For example, in 2020, Wang et al. synthesized poly(itaconic anhydride-*co*-vinyl caprolactam) (PIVC) with orange-red emission and poly(itaconic anhydride-*co*-vinylpyrrolidone) (PIVP) with bright white emission in solid powders by a radical precipitation copolymerization method (Figure 2A).^57^ This work confirmed that unconventional chromophores and increasing the flexibility of polymer chains are conducive to enhanced and red-shifted emission. In addition, simple molecular-dependent fluorescence patterns did not hold for different types of polymers. The differences in photoluminescence of the polymers are more likely to depend on whether their chemical and aggregation structures, and their final conformations, are conducive to strong physical interactions and TSI. The authors also discussed the fluorescence of homopolymers and copolymers. A suitable molecular chain conformation that is conducive to stronger intrachain and/or interchain interactions is the decisive factor in determining the emission of these NCLMs.^57^ At low concentrations, it is difficult for the polymer chains to be in close contact and to aggregate, and hence the interchain interactions are weak, so the emission of the copolymer is simply the emission of the added homopolymer. However, in a concentrated solution or solid state, large aggregates form, resulting in strong interchain/intrachain interactions, and the emission of the copolymer is not simply the addition of the parent homopolymers’ emission.

In 2022, Yue et al. synthesized a series of aliphatic hyperbranched polyureas (aBPUs) with different degrees of branching. The photophysical properties of the aBPUs are highly dependent on the chemical modification in their branches or interior: with increased branching, the fluorescence of aBPU is enhanced and a red-shift is observed. Finally, an aBPU-PEG nanoassembly was applied to *in vitro* labeling-free imaging of 4T1 murine carcinoma cells (Figure 2B).^67^ Zhang et al. obtained nonconjugated and nonaromatic polyesters (P1–P4) with variable luminescence colors and controllable efficiency through copolymerization of propylene epoxide and a cyclic anhydride. Different anhydrides were used to manipulate segmental flexibility and rigidity. Among them, polyester P3 has the longest luminescence wavelength and the highest QY (37.9%, solid state) (Figure 2C).^58^

**Figure 3:**
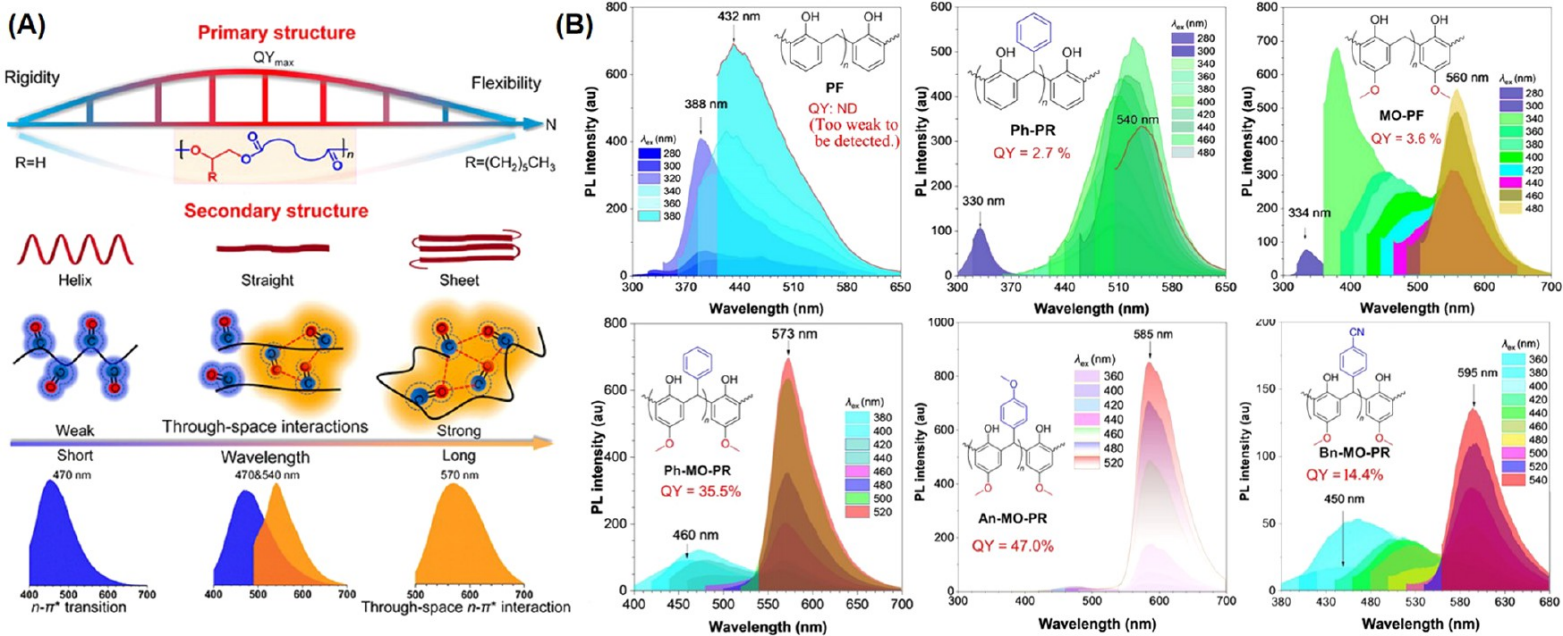
(A) Effects of primary and secondary structures on the photophysical properties of aliphatic polyesters. Red dotted lines represent the through-space interactions.^40^ Reprinted with permission from ref (40). Copyright 2022, American Chemical Society. (B) Chemical structures, QY and excitation-dependent spectrum of six phenolic resins in THF solutions.^52^ Reprinted with permission from ref (52). Copyright 2023, John Wiley and Sons.

Although TSI theory has greatly advanced the understanding of unconventional luminescence in the past few years, the structure–property relationships of TSI still need to be explored further. Zhang et al. found that like the extended TBC in π-conjugated photoclusters, hierarchical TSI plays a crucial role in nontraditional chromophores, similar to the multilevel structure of proteins: that is, higher levels of TSI within the molecule may contribute to longer-wavelength emission. Especially for hydrocarbon nontraditional chromophores, without strong donors and acceptors, the stable linking of multiple lower-level TSI units can bring higher-level TSI. Therefore, if intramolecular/intermolecular TSI can be controlled at the molecular level, this will be another breakthrough in the development of NCLMs.^37^ In 2022, Zhang et al. synthesized 24 nonconjugated aliphatic polyesters with tunable luminescence color and efficiency by copolymerization of six epoxides and four anhydrides.^40^ This was the first example of white luminescence achieved from a nonconjugated linear polyester. The experimental and computational results showed that the hierarchical structure plays an important role. At the primary level, balancing the flexibility and stiffness of the polyester chains is an effective and reliable method to improve the QY. The manipulation of the high-level structure produces partially stable carbonyl group clusters, improving the efficiency of luminescence (up to QY of 20.3%), and achieves long-wavelength luminescence (λ_max_ 570 nm) through strong inter/intrachain through-space n-π* interactions (Figure 3A).^40^ This work exemplifies a strategy for manipulating luminescence properties and implementing NCLMs by modulating the hierarchy.

In 2023, Tang et al. synthesized six phenolic resins with different substituents that emitted from deep-blue to the NIR region through a simple polycondensation reaction. On the one hand, increasing the number of TSI units promoted the appropriate structural conformation for TSIs and increased electron density leading to strong TSC or through-space locally excited states. On the other hand, the wavelength of luminescence was further increased by introducing the TSCT state from electron donating and electron-withdrawing groups. Ultimately, polymer An-MO-PR achieved bright NIR emission with λ_max_ at 680 nm, extending to 800 nm in the solid state, and λ_max_ at 585 nm in THF solution with a high QY of 47% (Figure 3B). This work reaffirms the importance of TSI and that increasing the electron density at the TSC center should be a more efficient strategy for producing both long wavelength and efficient luminescence, compared to building D–A structures.^52^

#### 2.2.3 Heating

**Figure 4:**
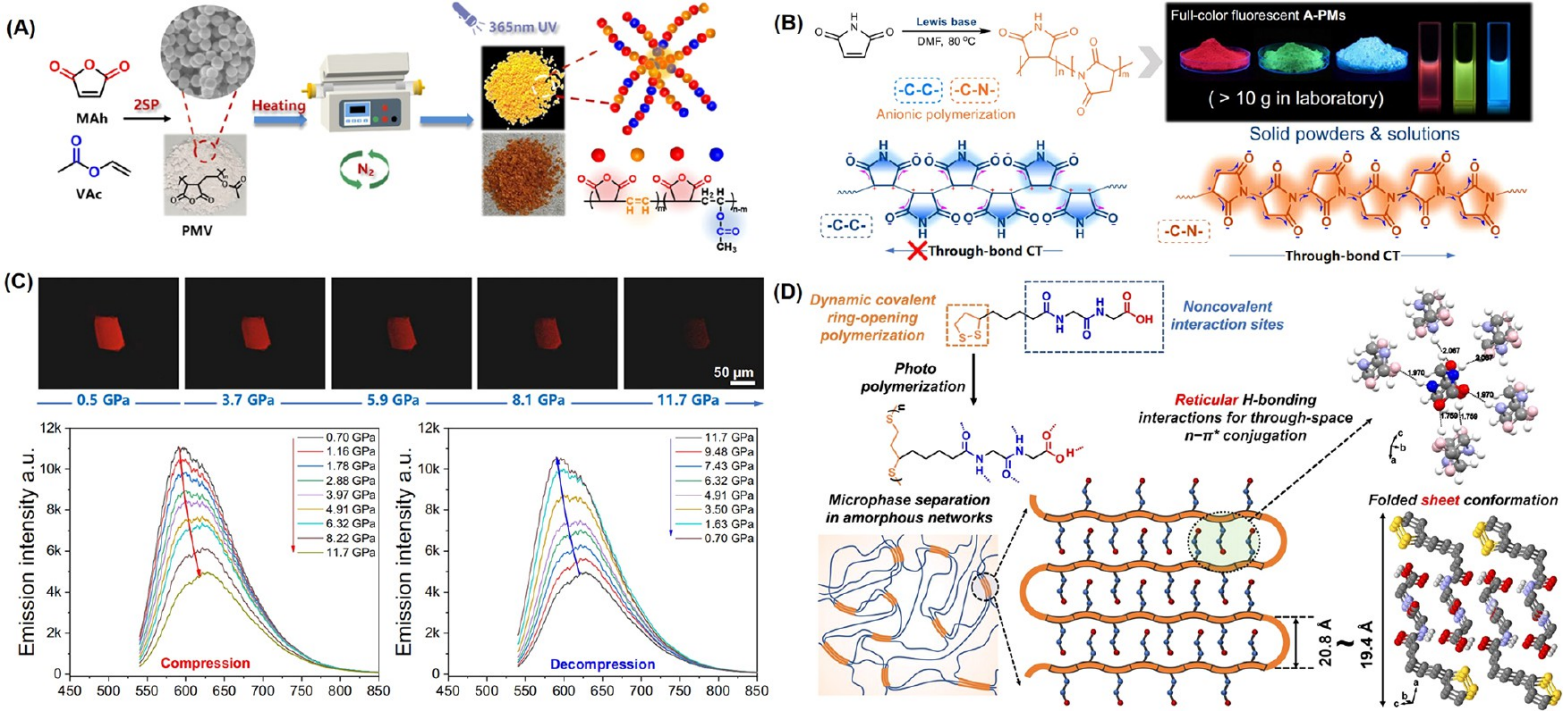
(A) Conceptual illustration of the protocols of heating PMV (2SP: self-stabilized precipitation polymerization method).^70^ Reprinted with permission from ref (70). Copyright 2023, American Chemical Society. (B) Molecular structures of the PMs in −C-C- and −C-N- connections and their full-color fluorescence photographs.^39^ Reprinted with permission from ref (39). Copyright 2022, Springer Nature. (C) In situ fluorescent images of A-PM-TEA compressed by different pressures; Emission spectra of A-PM-TEA during compression (0.70–11.70 GPa) and decompression (11.70–0.70 GPa).^39^ Reprinted with permission from ref (39). Copyright 2022, Springer Nature. (D) Conceptual illustration of the monomer molecular structure and the self-assembled polymeric networks.^73^ Reprinted with permission from ref (73). Copyright 2023, John Wiley and Sons.

Heating (including water and air heating) has been underestimated as a simple method to regulate luminescence of NCLMs. Wang et al. demonstrated that upon thermal treatment in air polyolefins without any chromophore can be transformed into fluorescent polymers. FTIR and XPS data showed that the oxidized polymers contained −OH, C=O and C–O groups appropriate for the clustering-triggered emission (CTE) mechanism.^68^ CTE is broad terminology used when heteroatoms (typically N, O, S or P) or unsaturated bonds (such as C=O, C=C, C≡N) aggregate into emissive clusters. The authors also developed a “gas-thermal method” to prepare NCLMs with enhanced and red-shifted fluorescence by heating weakly blue-emitting polymers [poly(vinyl alcohol), polyethylene glycol and starch] in different gas environments (air or nitrogen) which introduced C=O and C=C units. The emission of the products could be easily adjusted by the change of atmosphere, temperature and heating time, which is a new idea and a general method for the preparation of NCLMs.^69^ Qiao et al. reported a simple heating process to prepare red emitting poly(maleic anhydride-*alt*-vinyl acetate) (PMV) derivatives. Vinyl acetate (VAc) was first converted to C=C units, which promoted the movement of the polymer chains and partial cross-linking, thus regulated the through-space conjugation. Then by adjusting the heating temperature and time, tunable emission with λ_max_ in the range 620–675 nm was obtained (Figure 4A).^70^ Bryce et al. studied the effects of heating on the aggregation behavior of NCLMs based on a polyurethane derivative in different initial states (solution, powder and gel). Heating samples at 80 °C for a few hours and then at 120 °C achieved a fluorescence transition faster than by heating only at 120 °C, implying that the aggregation process requires time to occur. The many noncovalent interaction sites on the polyurethane chain were conducive to the aggregation-induced polychromatic blue-to-red fluorescence.^71^

### 2.3 Exploration of Full-Color Emission

Compared with traditional (π-conjugated) AIE-OLMs, the adjustment of emission color through rational molecular design combined with high emission efficiency is a major challenge that hinders the practical applications of nonconventional chromophores. Although most NCLMs have excitation-dependent luminescence properties, their emission windows are mostly limited to the 400–500 nm range. More precise guidelines are needed to accurately manipulate the intra/intermolecular interactions that ultimately regulate the macro-luminescence mechanism and color of NCLMs. Recent strategies are discussed in the following paragraphs.

#### 2.3.1 Aggregation Mode and Mechanism

Facilitation of TSCT or intermolecular charge transfer via spatially separated D–A groups on nontraditional polymer chains, or supramolecular self-assembly, can lead to red-shifted emission in NCLMs. For example, Zhang et al. achieved full-color range emission of nonconjugated poly(maleimide)s (PMs) by anionic polymerization of maleimide. The continuous D-A-D-A backbone structure greatly facilitates electron delocalization. The unconventional panchromatic emission of PMs originates from intra/intermolecular through-bond charge transfer (TBCT) or TSCT to different degrees, which is mainly determined by the bonding mode (−C-C- and −C-N-), molecular weight, and aggregation state in the polymerization process under different conditions (Figure 4B).^39^ The authors noted that general characteristics such as concentration-enhanced emission and excitation-dependent luminescence were not observed. Experiments under high pressure showed that shortening the distance between chains under compression promoted TSCT and intramolecular charge transfer. When the pressure increased from 0.7 to 11.7 GPa, the fluorescence intensity of polymaleimide decreased and the λ_max_^em^ was red-shifted. This was a reversible process (Figure 4C).^39^ The biocompatibility of the luminescent PM powders was exploited in proof-of-concept forensic identification based on interactions between the polar imides and fingerprint residues (e.g., proteins and amino acids).

By simply changing the initiators and free radical polymerization conditions Lin et al. prepared poly(acrylamide)s (PAMs) with tunable RTP and λ_max_^em^ in the range 470–550 nm and a maximum lifetime of τ_ph_ 361 ms.^72^ Qu et al. reported an oligopeptide-modified 1,2-dithiolane small-molecule which combined disulfide-mediated dynamic covalent ring-opening polymerization and reticular H-bond cross-linking, resulting in supramolecular networks with β-sheet H-bond linkages (Figure 4D).^73^ The electron-rich hard β-sheet domains promoted *n*-π* transitions for TSC and red-shifted the emission toward the green region (480 nm) and even phosphorescence (614 nm; lifetime τ_ph_ 3.40 ms) at 77 K. The synergy of dynamic poly(disulfides) and multiple H-bonding imparted mobility to the polymer network under mild conditions, including the self-healing of scratches on the polymer film surface.

Klajnert-Maculewicz et al. observed that attaching 1-(4-carbomethoxy)pyrrolidone (4-CMP) groups to the surface of poly(amidoamine) (PAMAM) dendrimers significantly increased their intrinsic blue fluorescence. This was explained as a consequence of two mechanisms: autofluorescence of the surface 4-CMP groups and an increase in emission from the interior due to dendrimer aggregation. With increasing dendrimer generation the penetration of a luminescence quencher into the dendrimer was impeded in line with critical nanoscale design parameters.^74^ Time-resolved fluorescence quenching studies employing a collisional quencher (methyl red) and a dynamic proximity quencher (nitrobenzoxadiazole dipeptide derivative) provided evidence for two spacially separated emission sites within this series of dendrimers.^75^

However, the limits of theoretical calculations of the electronic structure of unconventional systems have undoubtedly hindered the development of NCLMs. More advanced and accurate computational methods are needed to support the interaction analysis of NCLMs in excited states. Gaussian or dynamics simulation software have been mainly used to optimize the molecular structure of NCLMs, analyze their conformation, calculate their electronic structure and electron density distribution, ground state/excited state energy levels, spin–orbit coupling constants, highest occupied and lowest unoccupied molecular orbital (HOMO and LUMO) contours, and the inter/intrachain short interatomic contacts.^39,40,44,71^ Also, the unconventional luminescence of small molecules has inspired and served to help the analysis of interactions in NCLMs.^76^ For example, Tang et al. obtained a new type of cyclodextrins through modification of amino acids. The effects of intramolecular n-n spatial interactions, intermolecular n-π spatial interactions and hydrogen bonding are discussed in detail based on theoretical calculations.^77^ Zhang et al. synthesized the small-molecule *N*-stearoyl-hydroxyproline (L-C16-Hyp). DFT calculations and natural transition orbital analysis established that its luminescence comes from n-π* transitions localized on the tertiary amide and the adjacent carboxyl group in the L-C16-Hyp structure.^78^ Došlić et al. calculated the vibration-resolved absorption and fluorescence spectra of 1,4-diazabicyclo[2.2.2]octane (DABCO). A variety of electronic structure calculation methods and large base sets were used to prove that DABCO’s luminescence comes from the vibronic coupling of the one-photon forbidden transition between the ^1^A_1_′(n_+_3s) state and the electronic ground state.^79^ Kim et al. used molecular docking software to calculate the interactions of a model trimer of a nonconjugated heteroatom-containing spiropolymer with the MDM2 protein, confirming that aggregation leads to the luminescence.^80^

#### 2.3.2 Change of Microenvironment

The noncovalent interactions that are central to NCLMs are very susceptible to microenvironments which can be conveniently adjusted. For example, Zhang et al. easily modulated the multicolor luminescence of poly(methyl vinyl ether-*alt*-maleic anhydride) (PMVEMA) using a pH-controlled hydrolysis strategy. In contrast to the widely accepted mechanism of clustering-triggered emission (CTE), they emphasize the primary role of hydrated hydroxide (H_2_O·OH^–^). Steady-state and time-resolved spectral features determined that a “hydrated hydroxide complex assisted p-band intermediate state” (H_2_O·OH^–^-PBIS) occurs by the strong overlap of the p orbitals of the carbon-based and hydrated hydroxide O atoms with through-space electronic interactions. The dynamic nature of H_2_O·OH^–^-PBIS makes it susceptible to the microenvironment, especially to pH, so the emission color of PMVEMA was easily adjusted from blue to red by controlling the alkalinity in the hydrolysis process.^81^ Tang et al. were inspired by the color change that occurs when maleimide reacts with alkaline reagents. The maleimide polymer was produced by 1,8-diazabicyclo[5.4.0]undec-7-ene (DBU) as base acting both as a polymerization initiator and an external factor. By varying the amount of DBU the ratio of purple (λ_max_ 430 nm) to orange-red (λ_max_ 580 nm) emission was controlled, and white-light emission was achieved in DMSO by merging the two peaks.^82^

### 2.4 The Acquisition of Long Lifetime Luminescence

The development of NCLMs with fluorescence and long-life room temperature phosphorescence is not only of great significance to reveal the nature of the emission mechanism of NCLMs, but also to stimulate the development of new families of functional materials.^83−85,55^

#### 2.4.1 Delayed Fluorescence (DF) and Thermally Activated Delayed Fluorescence (TADF)

**Figure 5:**
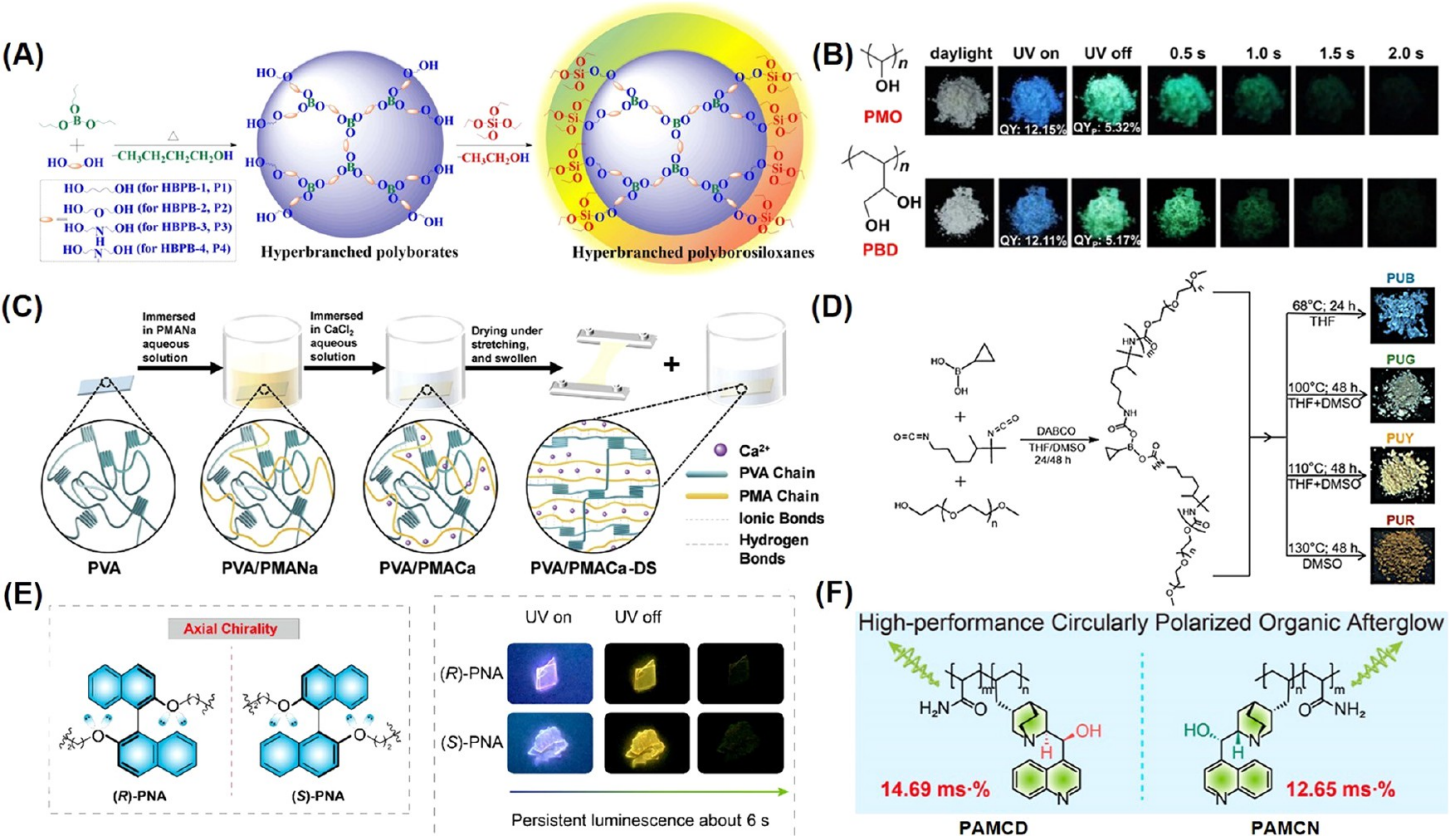
(A) Synthesis route to hyperbranched polyborosiloxanes.^84^ Reprinted with permission from ref (84). Copyright 2023. (B) Molecular structures and photographs of PMO and PBD taken in daylight, before and after ceasing the 365 nm UV irradiation.^53^ Reprinted with permission from ref (53). Copyright 2023, Elsevier. (C) Schematic illustration of the preparation process and physical interactions in the PVA, PVA/PMANa, PVA/PMACa, and PVA/PMACa-DS hydrogels.^89^ Reprinted with permission from ref (89). Copyright 2024, Wiley-VCH Verlag. (D) Synthesis of the polyurethanes and photographs of the corresponding powder samples under 365 nm illumination.^90^ Reprinted with permission from ref (90). Copyright 2024, Royal Society of Chemistry. (E) Molecular structures of (*R*)-PNA and (*S*)-PNA; photographs of (*R*)-PNA and (*S*)-PNA films taken under a 254 nm lamp on and off.^93^ Reprinted with permission from ref (93). Copyright 2021, Royal Society of Chemistry. (F) Molecular structures and FM values of PAMCD and PAMCN (FM = τ_PL_ × PLQY × |*g*_lum_|).^94^ Reprinted with permission from ref (94). Copyright 2023, American Chemical Society.

In 2021, Zhang et al. reported a series of thermally activated delayed fluorescence (TADF) polymers based on a combination π– conjugated donor–acceptor units and rigid nonconjugated polyimide linker units which have a key role to inhibit intramolecular charge transfer and endow high thermal stability. High-performance polymer light-emitting diodes (PLEDs) were realized with a maximum external quantum efficiency (EQ*E*_max_) > 21.0% and low efficiency attenuation over a large brightness range.^86^ In 2023, Xu et al. synthesized a series of copolymers with nonconjugated aliphatic backbones and pendent 9,9-dimethylacridine donor (D) and triazine-phosphonoxy acceptor (A) groups. The σ-linkage gives through-space charge transfer (TSCT) which can be optimized by control of the separation distance and relative orientation of the D and A units. The copolymer achieved synergies between electronic and spatial effects and had balanced and complementary intrachain and interchain TSCT. This led to smaller HOMO–LUMO overlap and a small energy gap between the lowest energy singlet (S) and triplet (T) states (Δ*E*_ST_) which are requirements for efficient reverse intersystem crossing (RISC) and TADF. The copolymer had high photoluminescence and electroluminescence quantum efficiency of EQ*E*_max_ 32.4%, with TADF performance.^51^ Yan et al. prepared a series of novel hyperbranched polyborosiloxanes (Figure 5A), with no traditional conjugated or aromatic groups, which achieved the tuning of unconventional light-emitting polymers from green to red by adjusting the electron density of monomer diol. The electron delocalization synergies induced by the excited Si and B atoms, as well as the strong TSI brought about by the through-space O···O and O···N ground-state interactions, reduced Δ*E*_ST_ (to 0.08 eV), promoted RISC, and ultimately gave long-lived delayed fluorescent (DF) red emission (τ 9.73 μs).^84^ The authors state that “the long-lived fluorescence of P4 resembles the DF observed in polycyclic aromatic hydrocarbons.” However, it was not established whether this DF is thermally activated. The polymers showed excellent potential for dual-information encryption based on fluorescence intensity and color variation under different excitation wavelengths.

#### 2.4.2 Room-Temperature Phosphorescence (RTP)

RTP requires strategies that overcome the spin-forbidden nature of excitation from the lowest singlet (S_1_) to triplet (T_1_) state, and the fast nonradiative decay of triplet excitons. This can be achieved by enhancing spin–orbit coupling and promoting intersystem crossing (ISC) by incorporation of aromatic carbonyls, multiple heteroatoms or heavy atoms (transition metals or halogens). For conventional AIE-OLMs, crystallization is an effective and common way to enhance RTP because it can restrict intramolecular motion to produce a rigid molecular conformation, which ultimately inhibits nonradiative decay. However, the difficulty of crystallizing polymers has limited many practical applications. In contrast, hydrogen bonding can be easily incorporated into amorphous polymers, as a crystallization-like strategy, to achieve conformational curing. For example, Yang et al. reported hydrogen bond-induced oxygen clusters in novel amorphous polyols with long-lived RTP. This NCLM system containing only oxygen atoms and σ electrons, and without crystallization achieved fluorescence QY of ca. 12% and phosphorescence (τ_ph_ 89 ms) at room temperature, through strong hydrogen bonding interactions and n-n interactions of oxygen atoms (Figure 5B).^53^ Fluorescence and long-lifetime RTP dual emission of polystyrene (styrene-*al*t-maleic anhydride) hydrolysate was achieved by introducing ionic and hydrogen bonds.^54^ It is worth noting that strong hydrogen bonding systems are prone to poor solubility, and overcross-linking is also not conducive to good solubility. Adjusting the conditions of the polymerization reaction and the post-treatment of products (such as freeze-drying) may solve this problem.

Lin et al. prepared a series of nonconjugated polysiloxane nanomaterials using different siloxanes as raw materials by a low-temperature solution method. Because the terminal amino or urea groups aggregate with silica and with the non-cross-linked hydroxyl groups to form suitable spatial clusters, the materials can emit bright blue and green afterglow, respectively, when the UV lamp is turned off. The QY of the amino-based nanomaterials reached 30.2% and a blue-green afterglow with a lifetime of 120 ms. When organic carbonyl-containing compounds were doped into the clusters full-color afterglow (λ_max_ 380–753 nm) was achieved.^87^ Wang et al. reported a series of nonaromatic amorphous polymers with yellow-orange-red RTP. Multiple nonaromatic donor–acceptor (nD-A) structures with carboxylate and lactam groups of different electron donor/acceptor capabilities, combined with short contacts, aggregation, and efficient TSCT, collectively suppressed nonradiative decay, resulting in a reduction in the energy gap and an increase in the probability of intersystem crossing (ISC), and ultimately strong, orange-red RTP at ≈600 nm, with τ_ph_ 41 ms.^88^

Luminous hydrogels/organogels based on NCLMs are still rare and their mechanical properties are largely unexplored although they offer great potential for the development and applications of soft materials. Wang et al. prepared high-strength hydrogels with RTP emission based on nonaromatic polymers. The preparation method is shown in Figure 5C. By introducing calcium ions and increasing the cross-linking density of ionic bonds and hydrogen bonds to create strong interchain interactions, the poly(vinyl alcohol) (PVA)/poly(calcium maleate) PVA/PMACa-DS hydrogels (where DS means dried under stretching and equilibrium swelling) have excellent mechanical properties with high tensile strength. Under excitation with 312–400 nm light, multicolor phosphorescence was observed from blue to yellow-green with a maximum τ_ph_ of 13.4 ms.^89^

A polyurethane derivative incorporating cyclopropylboronic ester units in the backbone is a molecular-weight dependent polychromatic clusteroluminescent material (Figure 5D). PUY attains an RTP lifetime of 0.45 s, representing the longest lifetime for a pure linear NCLP reported to date.^90^

#### 2.4.3 Circularly Polarized Phosphorescence (CPP)

NCLMs that emit circularly polarized luminescence (CPL) have rich structural variety, simple manufacture, high thermal stability, and adjustable performance. CPL broadens the applications of NCLMs in the fields of chiral recognition sensors, noninvasive biomedical diagnostics, and catalysts for asymmetric synthesis.^91^ Circularly polarized phosphorescence (CPP) has particular applications in OLEDs and encryption displays.^92^ Zhao et al. realized CPP from isolated chromophores with axial chiral characteristics in polymer chains. Polyacrylic acid was chosen as the polymer matrix because its many carboxyl groups facilitate the ISC process to produce triplet excitons and to reduce nonradiative decay by building a rigid network that effectively immobilizes the phosphor. Circularly polarized fluorescent and phosphorescent dual emission were obtained from the films of brominated derivatives of (*R*)-PNA and (*S*)-PNA, while (*R*)-PNA and (*S*)-PNA films had yellow afterglow lasting about 6 s (Figure 5E).^93^ Chen et al. used naturally occurring chiral cinchonine copolymerized with acrylamide and subjected to aggregation regulation by dissolution in water and thermal evaporation to achieve high-quality CP organic afterglow from the nonconjugated copolymer PAMCD and its enantiomer PAMCN (Figure 5F). Excitation-dependent encryption and reversible anticounterfeiting devices with high stability were demonstrated.^94^

### 2.5 High Efficiency Emission

Due to the lack of in-depth understanding of the luminescence mechanism and the difficulty in regulating the aggregates’ structure, most of the reported QYs of NCLMs are low (<20%) which hinders their practical applications. Especially, how to combine long-wavelength emission with high QY is a topical issue that needs further extensive studies to advance the theory of aggregate photophysics and to develop more efficient NCLMs.

**Figure 6:**
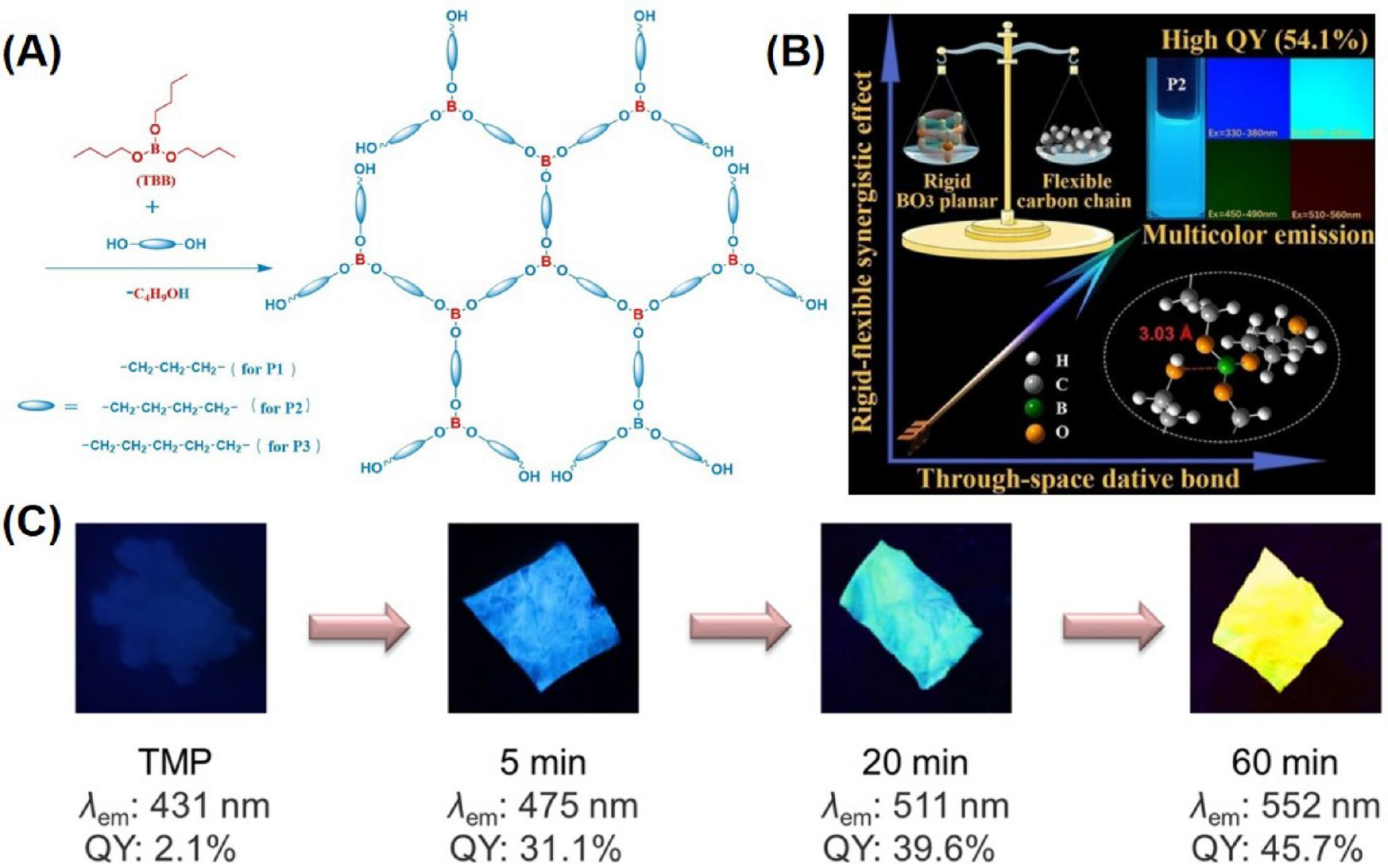
(A) Synthetic routes of hyperbranched polyborates (P1–P3).^44^ Reprinted with permission from ref (44). Copyright 2022, John Wiley and Sons. (B) Schematic diagram of regulation mechanism of high-QY hyperbranched polyborates.^44^ Reprinted with permission from ref (44). Copyright 2022, John Wiley and Sons. (C) The photographs of TMP and polymeric films at different polymerization times under UV light.^95^ Reprinted with permission from ref (95). Copyright 2023, Springer Nature.

An appropriate chain structure is needed to optimize the polymers’ rigidity while retaining sufficient conformational flexibility to ensure the formation of close aggregates. Although most NCLMs incorporate multiple electron-rich heteroatoms, derivatives with electron-deficient boron atoms have also received attention. For example, Yan et al. prepared three high-QY hyperbranched polyborates using tributyl borate and diols with different carbon chain lengths (Figure 6A). The absolute fluorescence quantum yield of P2 is as high as 54.1%, which is comparable to some aromatic polymers. The rigid BO_3_ plane limits the movement of molecular segments, while the flexible aliphatic chains allow intermolecular aggregation. The authors propose that the synergistic “rigid and soft effect” can effectively reduce nonradiative transitions. The electron-rich oxygen atoms, and the boron atoms with their vacant p orbital, form a “spatial coordination bond” which promotes the exchange of charges within the aggregate (Figure 6B).^44^ Tang et al. obtained a self-supporting polymer film through polymerization of a nonaromatic terminal tri(yne-ester) monomer (TMP) involving water. The reaction involves an interesting “interfacial polymerization-induced luminescence enhancement” phenomenon: with the extension of reaction time, the luminescence of the polymer film red-shifted (blue → green → yellow) and the luminous efficiency also sequentially improved. After 1 h of reaction, the film emitted bright yellow light, with a PLQY as high as 45.7% (Figure 6C).^95^

### 2.6 Applications

Due to their rich structural diversity, low synthetic cost, good mechanical properties, easy processing and sensitivity to the microenvironment, NCLMs have been widely used in multidimensional information encryption/anticounterfeiting, optoelectronic devices, fingerprint recognition, detection of metal ions and volatile solvent vapors, biological analysis and imaging, multicolor ink printing and other fields.^95−98,30^

#### 2.6.1 Anti-Counterfeiting and Encryption

**Figure 7:**
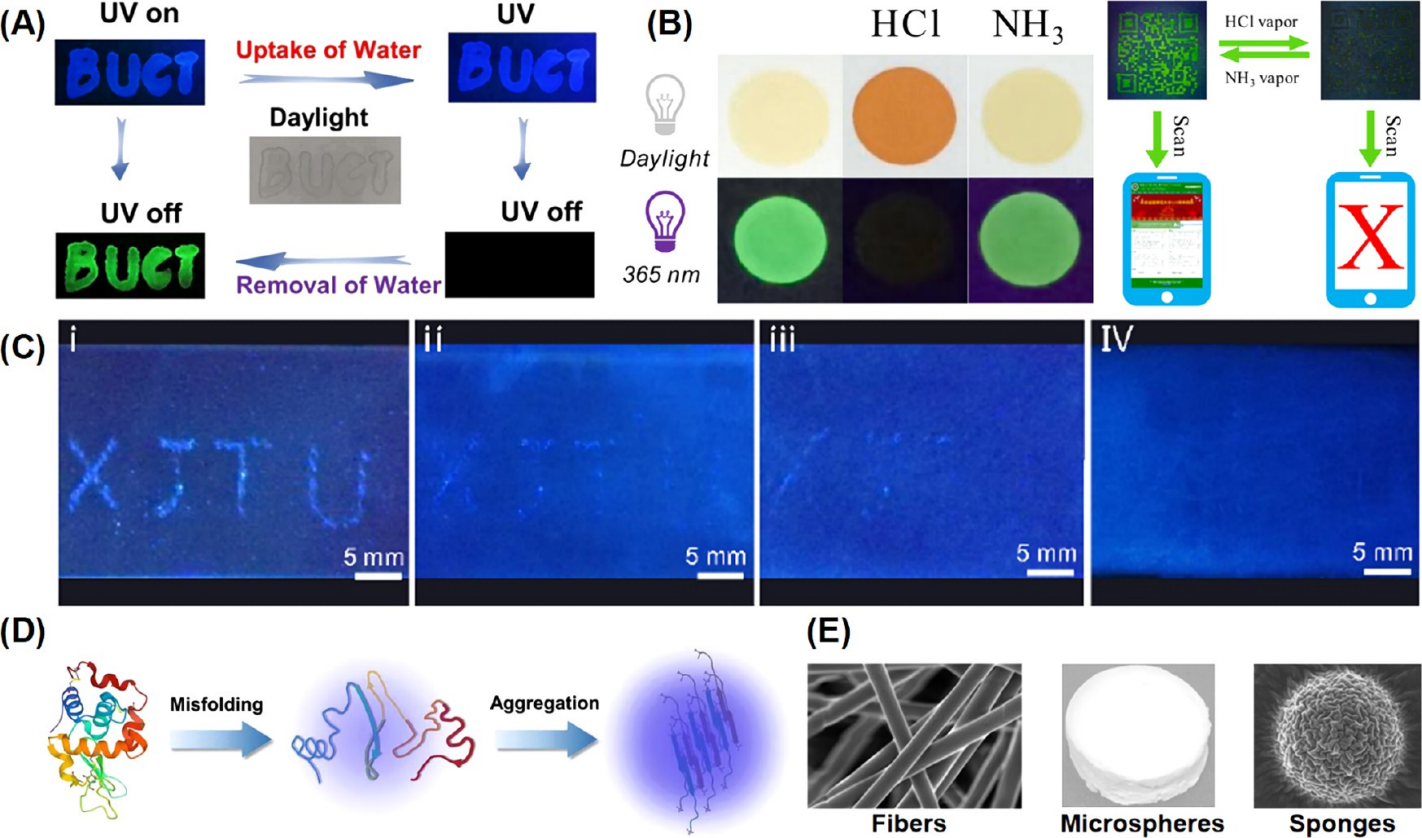
(A) Photographs of letters “BUCT” using NCLMs as the ink under daylight, 365 nm UV-on, and UV-off.^19^ Reprinted with permission from ref (19). Copyright 2021, American Chemical Society. (B) Digital photos of PDMATPM upon acid (HCl vapor)/base (NH_3_ vapor) (under daylight and UV lamp @365 nm); Photographs of a QR code-based luminescent encryption and decryption upon HCl and NH_3_ vapor.^101^ Reprinted with permission from ref (101). Copyright 2023, Elsevier BV. (C) Crack shaped XJTU on the healable surface of HPDU film healed for (i) 0 h, (ii) 3 h, (iii) 6 h, and (iv) 12 h at 90 °C.^107^ Reprinted with permission from ref (107). Copyright 2020, American Chemical Society. (D) Schematic illustration of the process of protein aggregation monitored by NCLMs.^109^ Reprinted with permission from ref (109). Copyright 2023, American Chemical Society. (E) Morphologies of PVC fibers, microspheres and sponges.^118^ Reprinted with permission from ref (118). Copyright 2023, Royal Society of Chemistry.

Ni et al. reported a blue-fluorescent aliphatic polyhydroxyurethane with shape memory and self-healing properties and exploited its unique characteristics to develop a method for “light-mediated ink-free screen printing” for the manufacture of anticounterfeiting paper. Broad applications are envisaged in the pharmaceutical, packaging and food industries.^99^ Deng et al. utilized a hydrolysis process to achieve ultralong RTP (τ_ph_ up to 400 ms) in biobased nonconjugated polymers comprising alternating dimethoxyphenylpropene and maleic anhydride monomer units. Hydrolysis of the anhydride units, which turned on the RTP, was reversible through water absorption and removal; different emission centers for unconventional luminescence and a wide range of RTP emissions led to anticounterfeiting applications (Figure 7A).^19^ Liu et al. prepared blends and random copolymers containing acrylic acid and acrylamide units with RTP efficiency of 12.0% and successfully applied them to information encryption. The work emphasized intermolecular H-bonding as the mechanism behind the RTP with high humidity resistance.^100^ Wan et al. prepared poly[(4-dimethylamino)triphenyl-methanol] (PDMATPM) with AIE properties and reversible acid/base responsive luminescence, and potential applications in luminescence encryption and decryption (Figure 7B).^101^ Yang et al. prepared an amide-terminated hyperbranched polyether which was cross-linked with boric acid to give a material with ultralong RTP (τ_ph_ 2.40 s; QY 24.6%) and significant temperature and humidity response. The polymer was applied to information encryption.^55^

#### 2.6.2 Sensing and Detection

The ability of NCLMs to respond characteristically to external stimuli or reagents offers many applications in monitoring and sensing, visualization, detection of physical transformations, etc. For example, Singha et al. prepared two aliphatic backbone polymers with pendent amide and ester substituents. Quenching of the polymers’ fluorescence upon metal ion coordination enabled the selective and sensitive detection and removal of Cu^2+^ and Fe^3+^ in water media. Reversible absorption/desorption cycles were demonstrated.^102^ Gu et al. reported the detection of Fe^3+^ and the explosive 2,4-dinitrotoluene by quenching of the blue fluorescence of a waterborne polyurethane derivative incorporating side-chain carboxyl groups. A higher carboxyl content increased the extent of H-bonding which rigidified the conformation, leading to oxygen-based clusterluminescence.^103^ Qin et al. prepared a series of biobased furfural polyamides and demonstrated their use as probes for the selective recognition of Fe^2+^ and Fe^3+^ in aqueous solution with high sensitivity compared to other metal ions, by a fluorescence quenching effect.^104^ Han et al. used bovine serum albumin as a natural protein cluster fluorescent probe to achieve a simple, rapid, sensitive, efficient and selective detection of the essential micronutrient ascorbic acid (vitamin C).^105^ Zhang et al. utilized nonconjugated poly(maleimide)s (PMs) for fingerprint detection by sensing proteins and amino acids.^39^

As the luminescence characteristics of NCLMs are intimately related to the molecular-scale motion and aggregation state they can be used as efficient fluorescence detectors to visualize the polymerization/emission process. For example, Qiao et al. introduced thioctic acid into the poly(maleic anhydride-*alt*-vinyl acetate) side chain, obtaining a dynamically cross-linked film with self-healing and reprocessable properties by volatilizing the solvent in an alkaline solution. Subsequently, relying on the dependence of cluster luminescence on aggregation distance, the aggregation distance was altered through the water uptake-loss equilibrium of carboxylic sodium groups at different humidities, achieving a humidity-responsive emission wavelength in the cluster luminescence (534–508 nm).^106^

#### 2.6.3 Crack Detection and Self-healing

Zhang et al. designed a mechanically tough, antipuncture, hyperbranched polyurethane (HPDU) elastomer incorporating diazolidinyl urea units to enhance the mechanical properties. The intrinsic blue fluorescence of the HPDU films enabled cracks to be identified using a UV light pen. Thermally induced diffusion of the polymer chains and H-bonding interactions in the HPDU films brought about self-healing of a cut film at 90 °C, which was monitored by a gradual decrease of the emission intensity (Figure 7C). The combination of low-cost crack diagnosis and self-healing is important for the long-term applications of polymers.^107^ Yang et al. used the sensitivity of hydrogen bonding to water to encapsulate cross-linked NCLMs in epoxy resin for crack detection based on phosphorescence behavior. Compared with traditional testing methods, this strategy greatly simplifies the detection process and reduces the technical barriers of the main current techniques.^108^

#### 2.6.4 Bioanalysis and Imaging

The good biocompatibility of NCLMs gives them great potential in biomedical fields such as cell imaging, formulation and sustained release of bioactive agents, and biosensors. For example, Zhang et al. explored the cluster luminescence characteristics of egg lysozyme and bovine serum albumin, and applied cluster luminescence to the simple monitoring of protein aggregation processes which are crucial markers in many human diseases (Figure 7D).^109^ De et al. developed a biocompatible fluorescence thermometer based on the heat-assisted AIE activity of a nonconjugated poly(*N*-vinylcaprolactam) (PNVCL) for intracellular temperature imaging in breast cancer cells (MCF-7). The key feature of PNVCL is bright blue emission above its lower critical solution temperature of 37.5 °C in aqueous media, due to a coil to globular conformational transition. Detecting minor temperature changes are beneficial for early detection and treatment of diseases and local hyperthermia.^110^ Singha et al. reported aliphatic intrinsically fluorescent terpolymers incorporating multiple pendent secondary amide groups that were attached *in situ* to induce AIE. A variety of applications were demonstrated, including selective sensing of Cr(III) (which has many important cellular functions) and *in vitro* imaging of human osteosarcoma cells.^111^ Kim et al. used the highly expressed MDM2 protein in tumor cells to bind to nonconjugated spiro polymers, and at the same time obtained nontraditional luminescence, restricting the binding of tumor suppressor protein (p53) to MDM2 protein, thereby releasing and activating p53 to achieve targeted diagnosis and *in vitro* apoptosis of tumor cells, without appreciable cytotoxicity in noncancerous cells.^80^ Ding et al. investigated peptidomimetic polyurea derivatives with AIE characteristics for conformation-assisted deformation, discoloration, and intracellular drug delivery with effective cancer theranosis *in vitro* and *in vivo*.^112^ Deng et al. prepared a series of polyamide derivatives (PAMs) containing pendent morpholine groups, which endow polymerization-induced emission, and applied them to target imaging trackers and as real-time monitors of changes of Fe^3+^ concentration in lysosomes.^113^ Tomalia, Klajnert-Maculewicz et al. showed that a functionalized PAMAM dendrimer forms a polyplex with double stranded DNA and is nontoxic for HeLa and HMEC-1 cells up to a concentration of 10 mg/mL. The unique intrinsic luminescence properties of the dendrimer revealed that it accumulates in endosomal compartments. The authors suggested that this tecto(dendrimer) could be an efficient transfection agent.^114^

#### 2.6.5 Lighting and Optoelectronic Displays

Electronic materials and devices for stretchable displays is currently a hot topic.^115^ Stretching a conventional light-emitting polymer which has one-dimensional π-conjugated chains is typically accompanied by a decrease in charge transport and hence a decrease in device efficiency. However, blends with polymers that have good chain flexibility offer outstanding potential in the field of optoelectronic devices. For example, Liu et al. developed a self-assembled three-dimensional penetration nanonetwork of high molecular weight poly(*p*-phenylenevinylene) (L-SY-PPV) (“super yellow”) and polyacrylonitrile to improve both tensile capacity and mobility. A PLED with 40% stretched organic layers had current efficiency of 8.13 cd A^–1^ and EQE 2.64%.^116^ In a separate study the PPV derivative was blended with polystyrene-*block*-polybutadiene-*block*-polystyrene elastomers to give a PLED which maintained 50% of its maximum luminance upon stretching by 60% of its initial length.^117^ It should be noted that in both of the above examples the emission was derived exclusively from the PPV component. NCLMs also bring some new opportunities for materials development. For example: Zhang et al. prepared electrospun fibers, electrospray microspheres, and sponges from poly(cyclic carbonate)s where clusteroluminescence is derived from intra- and intermolecular TSI of the oxygen atoms (Figure 7E).^118^ Qiao et al. used poly(methyl vinyl ether) derivatives for the development of novel agricultural films that convert ultraviolet light and unwanted green light into blue (400–500 nm) or red (600–700 nm) to increase the yield of photosynthetic crops.^70^

## 3 Challenges and Prospects

This Perspective has highlighted progress since the start of 2020 in NCLMs whose remarkable luminescent properties do not rely on classical chromophores. The emerging potential for (bio)degradation of some of these materials into environmentally benign components is an attractive attribute.^119,120^ Compared with classical AIE-OLMs, NCLMs that rely on intrinsic luminescence have the advantages of lower synthesis cost, excellent processability, wide availability in drug delivery, optoelectronic devices, and other fields.^1,65,121^ With the involvement of a diverse range of scientists, remarkable progress has been made in the basic understanding of the molecular design and photophysical properties of NCLMs. However, we are still some distance from the establishment of a complete nontraditional photophysical theory. In recent years, methods such as dynamic light scattering, XPS, rheological tests and high-pressure combined with fluorescence/infrared spectroscopy, molecular dynamics simulations, transient absorption, theoretical calculations, etc. have helped to decipher the emission sources of NCLMs.^122,123^ Compared with the traditional AIE emission behavior which relies on inhibition of molecular motion the intrinsic luminescence of NCLMs pays more attention to the spatial electron delocalization of the whole molecule. This electron delocalization refers to the conjugation of electron clouds in space through the synergistic effect of polymerization/aggregation/conformational rigidification in a broad sense. More importantly, compared with small molecules, the soft segment structure and more abundant conformational changes of macromolecules give them enhanced potential for interesting photophysical properties. It can be expected that new and unexpected photophysical phenomena will be discovered through innovative molecular designs and deeper experimental and computational characterization, leading to more practical and commercial applications of NCLMs. The exciting future development of NCLMs should mainly focus on the following aspects: (i) Exploring new materials with unconventional chromophore combinations. (ii) Finding suitable simplified models and more advanced computing techniques to dig deeper into the luminescence mechanism. (iii) Exploiting advanced intelligent applications based on NCLMs. (iv) Obtaining explicit relationships between molecular structure and intrinsic luminescence behavior, which is of great significance for the design and synthesis of NCLMs with high PLQY. (v) Continuing to explore phosphorescent NCLMs with adjustable luminous colors. (vi) Developing new NCLMs with CPL properties and establishing unified evaluation criteria for the design of such materials. Progress in these areas is eagerly awaited!
